# Extreme Short Stature and Severe Neurological Impairment in a 17-Year-Old Male With Untreated Combined Pituitary Hormone Deficiency Due to *POU1F1* Mutation

**DOI:** 10.3389/fendo.2019.00381

**Published:** 2019-06-27

**Authors:** Hussein Majdoub, Serge Amselem, Marie Legendre, Shoshana Rath, Dani Bercovich, Yardena Tenenbaum-Rakover

**Affiliations:** ^1^Pediatric Endocrine Clinic, Clalit Health Services, Northern region, Haifa, Israel; ^2^Sorbonne Université, Inserm U933 and Assistance Publique de Hopitaux de Paris, Hôpital Trousseau, Paris, France; ^3^Ha'Emek Medical Center, Pediatric Endocrine Institute, Afula, Israel; ^4^Tel Hai College and GGA - Galilee Genetic Analysis Lab, Katzrin, Israel; ^5^Rappaport Faculty of Medicine, Technion, Haifa, Israel

**Keywords:** POU1F1 gene, GH deficiency, GHD, central hypothyroidism, congenital hypothyroidism, CH

## Abstract

**Background:** POU1F1 is an essential transcription factor for the differentiation, proliferation and survival of somatotrophs, lactotrophs, and thyrotrophs. Mutations in the *POU1F1* gene are characterized by growth hormone (GH), thyrotropin, and prolactin deficiencies, commonly presenting with growth retardation and central hypothyroidism. Since the first report in 1992, more than 25 mutations have been identified in *POU1F1*.

**Case Description:** We describe a 17-year-old male who presented to our Pediatric Endocrinology clinic with extreme short stature (height 81.7 cm, −9.3 SD), cognitive impairment, deaf-mutism, and neurological disabilities. L-thyroxine supplemental therapy, which had been initiated at the age of 6 months but ceased due to non-compliance, was reintroduced at presentation. GH therapy was initiated at 19 years of age, resulting in 42 cm linear growth, to a final height of 124 cm. Sequencing of *POU1F1* revealed a previously described homozygous insertion mutation—c.580_581insT, p (Thr194Ilefs^*^7)—in exon 4 causing a frameshift that introduces a stop codon 7 amino acids downstream, leading to a severely truncated protein lacking the homeodomain.

**Conclusion:** This case report sheds light on the natural history of untreated patients with *POU1F1* mutations and raises awareness for early diagnosis and adequate treatment of central congenital hypothyroidism and GH deficiency.

## Introduction

Combined pituitary hormone deficiency (CPHD) is a rare heterogeneous condition in which there are at least two anterior pituitary hormone deficiencies. Anterior pituitary-derived hormones require multiple transcription factors for pituitary morphogenesis, differentiation and hormones synthesis. Mutations in the genes *PROP1, POU1F1 (PIT1), LHX3, GLI2, HESX1, LHX3, LHX4, OTX2, SOX2, ROBO1*, and *SOX3* (1–5), which encode transcription factors essential for pituitary development, have been described in human CPHD with an incidence of between 1:4,000 and 1:8,000.

POU class 1 homeobox 1 (POU1F1), also known as pituitary transcription factor 1 (PIT-1), was the first pituitary-specific transcription factor to be identified in humans and mice (6, 7). It is expressed in the anterior pituitary and is essential for differentiation, proliferation and survival of somatotrophic, lactotrophic, and thyrotrophic cells.

The human *POUIFI* gene (OMIM 601538) is located on chromosome 3p11, consists of 6 exons and encodes a 317-amino acid protein (NM_001122757) that has three functional domains: a transactivation domain, a POU-specific (POU-S) domain and a POU-homeo (POU-H) domain. The N-terminal part of POU1F1 is involved in the transcriptional activation of several pituitary-expressed genes (7). The first mutations of *POU1F1* in humans were described in 1992 (8–10), involving growth hormone (GH), prolactin (PRL), and thyrotropin (TSH) deficiencies. Since those first reports, more than 25 *POU1F1* mutations characterized as having clinical significance and presenting with CPHD have been described (https://www.ncbi.nlm.nih.gov/clinvar?term=173110[MIM]); these mutations are responsible for dominant or recessive CPHD, with 3 small insertion mutations among them (11–32) The clinical characteristics of patients with *POU1F1* mutations include severe growth retardation in infancy due to GH deficiency (GHD) with distinctive facial features characterized by prominent forehead, marked mid-facial hypoplasia, and depressed nasal bridge. In addition, central hypothyroidism of variable severity has been reported, and brain magnetic resonance imaging (MRI) demonstrates either normal or hypoplastic anterior pituitary with unaffected posterior pituitary.

Here we describe a 17-year-old male who presented with extreme short stature, mental retardation, deaf-mutism, neurological impairment, and dysmorphic features, in whom CPHD (GH, TSH, and PRL deficiencies) caused by a previously described homozygous frameshift mutation of the *POU1F1* gene (30) was identified. Marked clinical improvement occurred with GH and levothyroxine (LT_4_) therapy. This description extends the recognized phenotype of *POU1F1* mutations, shedding light on the severity of presentation in the untreated state.

## Case Presentation

**Figure 1:**
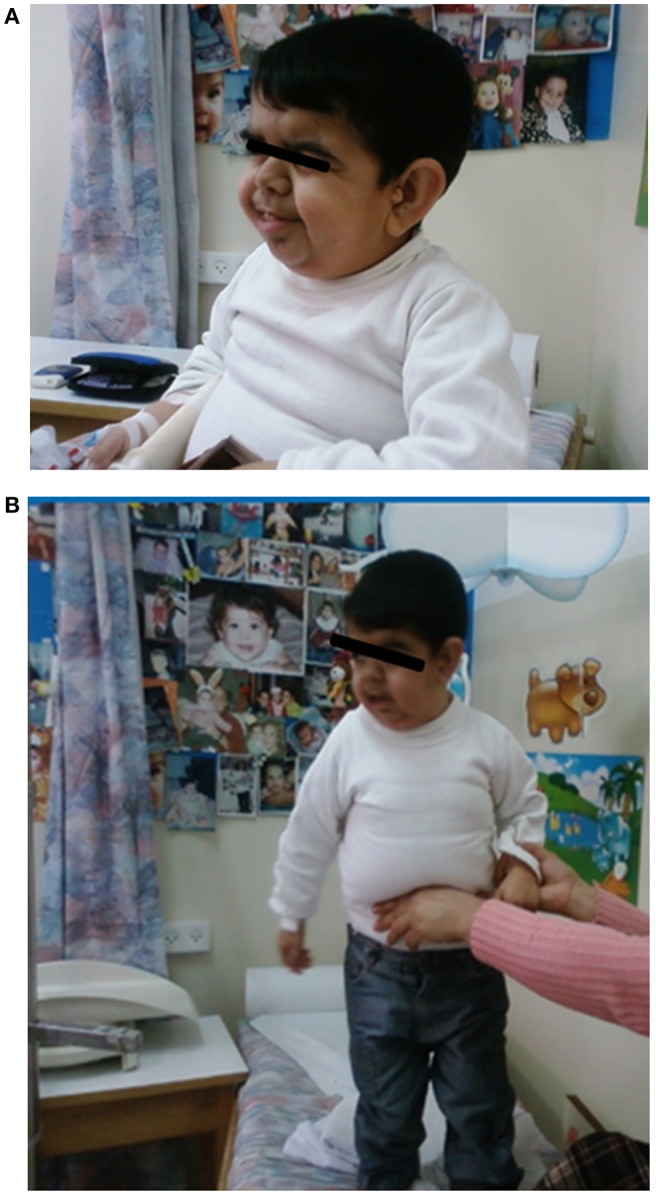
The patient at age 17 years. He had a deep nasal bridge, prominent forehead, micrognathia, flat nose, prominent and large auricles, multiple pre-auricular skin tags, and widely spaced teeth (With permission from the family).

**Figure 2:**
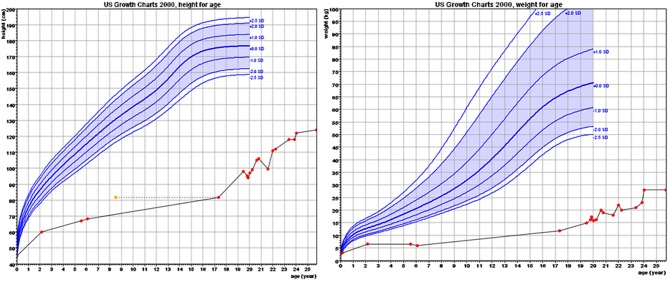
Growth charts of height and weight.

The proband, a male born to consanguineous parents (first cousins) of Arab descent, was born after pregnancy with polyhydramnios and delivered at term, weighing 2,900 g and his length was 45 cm. Both parents were healthy; paternal height was 170 cm and maternal height 160 cm. There was one healthy brother of normal stature. There was no family history of short stature, hypothyroidism, deafness, or neurological impairment. In the first week of life, the proband had recurrent vomiting and prolonged neonatal jaundice with indirect hyperbilirubinemia of 15 mg/dL that resolved spontaneously. At age 6 months, severe failure to thrive and dehydration were diagnosed, resulting in hospital admission. Delayed psychomotor development, growth retardation, and dysmorphic facial features were noted. On physical examination, he had enlarged fontanelle, severe hypotonia, umbilical hernia, and dry skin consistent clinically with hypothyroidism. Laboratory evaluation revealed low TSH (0.08 mIU/L; normal range 0.27–4.2) and free thyroxine (FT_4_) (5.4 pmol/L; normal range 12–22), indicating central hypothyroidism. Further investigation demonstrated mild bilateral frontal and interhemispheric cortical atrophy (on brain computed tomography (CT) scan and sensorineural (SN) hearing loss (Brainstem Auditory Evoked Response test). Comprehensive metabolic investigations were normal, as were echocardiography and ophthalmic examination. The karyotype was 46, XY, and no chromosomal abnormalities were identified. LT_4_ treatment was initiated at a dose of 30 μg daily, but was discontinued by the parents after 2 months. At the age of 4 years, he was diagnosed with severe mental retardation and was fully disabled. At 5 years of age, the proband presented to a different Endocrine clinic for investigation of severe growth retardation. At that time, TSH was 0.01 mU/L, FT_4_ 1.34 pmol/L and FT_3_ < 0.44 nmol/L (normal range 1.3–3.1). Thyrotropin-releasing hormone (TRH) test confirmed the diagnosis of central hypothyroidism (Table 1) and LT_4_ therapy was recommended. In addition, insulin growth factor-1 (IGF-1) and insulin growth factor binding protein-3 (IGFBP-3) levels were undetectable with no GH response to arginine stimulation test (Table 1). Unfortunately, although the family was informed on several occasions as to the importance of supplemental LT4 therapy and the need for GH treatment, compliance remained poor and the family failed to attend follow-up appointments so GH therapy was not initiated. The patient was seen for the first time in our Pediatric Endocrine clinic when he presented at age 17.5 years for evaluation of extreme short stature. At that time, his appearance resembled that of a 2-year-old child. His height was 81.7 cm (−9.3 SD) and weight 11.5 kg (−9.3 SD). He had dysmorphic facial features with a deep nasal bridge, a prominent forehead, micrognathia, flat nose, big, prominent auricles, multiple preauricular skin tags, and widely spaced teeth (Figures 1A,B). He had acromicria and a prominent abdomen without organomegaly. His pubertal stage was Tanner 1 with testicular volume of 2 mL bilaterally. Severe neurodevelopmental delay was noted, with general hypotonia; he was unable to stand unsupported. He was deaf and mute, communicating with sign language at a basic level. Investigations revealed a bone age of 8.5 years (at chronological age 17.5 years), undetectable TSH, FT_4_ 3.8 pmol/L, PRL < 8 mIU/L (normal range 45–375), IGF-1 < 3.25 ng/mL, and IGFBP-3 < 500 ng/mL. Cortisol and adrenocorticotropic hormone (ACTH) levels were within normal limits and gonadotropin levels were pre-pubertal (Table 1). As previous investigations were unavailable at that time, the diagnoses of central hypothyroidism and GHD were reconfirmed by stimulation tests. Brain MRI demonstrated a hypoplastic anterior pituitary, a normally located posterior pituitary bright spot, white matter demyelination and cervicomedullary kinking. Biochemistry, celiac screen, and complete blood count were within normal limits. LT_4_ therapy was recommenced with dose adjusted to maintain FT_4_ within a normal range. GH therapy was initiated with dose adjusted to weight (0.03 mg/kg per day) and growth velocity. Gonadotropin-releasing hormone (GnRH) analog treatment (3.75 mg every 28 days) was initiated at the onset of puberty (Tanner P2, G2, age 21 years) to facilitate maximal linear growth. Calcium supplement and vitamin D_3_ were added due to low serum levels of vitamin D. In addition, physiotherapy and speech therapy were implemented. At the age of 24 years, growth velocity decreased to <2 cm per year and therefore GH and GnRH analog therapy were terminated. At 28 years of age, height had increased by 42.3 cm (+3 SD) to a final adult height of 124 cm (−6.2 SD; Figure 2), and weight was 28 kg (−7.5 SD). Pubertal stage Tanner P5 was attained with testicular volume of 15 mL bilaterally. Considerable improvement in tone and psychomotor function was observed consequent to implementation of appropriate therapy; he learned to stand independently, walk several steps unsupported, and communicate vocally with family and medical staff. Overall, he had a happier demeanor, but unfortunately remained non-verbal.

**Table 1:** Summary of hormonal results.

**Test**	**Age (years)**	**0.5**	**5**	**17.5**	**25.10**	**Normal range**
	TSH (mIU/L)	0.08	0.011	<0.015	<0.015	0.4–4.2
	Prolactin (mIU/L)		<44	<0.8	<3.0	45–375
	FT4 (pmol/L)	5.4	1.34	9.5	20.8	10–20
	FT3 (pmol/L)		<0.044		7.4	3.5–6.3
TRH	Peak TSH		0.014	<0.015		
	Peak prolactin (mIU/L)		<44	<0.8		
	IGF-I (ng/mL)		<0.04	<3.25	<3.0	93–250
	IGFBP-3 (ng/mL)		<200	<500		2,700–8,900
Arginine	Basal GH (ng/mL)		<0.5			
	Peak GH /(ng/mL)		<0.5			>7.5
Clonidine	Basal GH (ng/mL)			<0.05		
	Peak GH (ng/mL)			<0.05		>7.5
Glucagon	Basal GH (ng/mL)			<0.05		
	Peak GH (ng/mL)			<0.05		
ACTH	Basal cortisol (nmol/L)		361	238	480	200–700
	Peak cortisol (nmol/L)		1,405			>550
	Basal ACTH (pmol/L)			2.53		0–10
GnRH	Basal LH (mIU/L)		<0.5	1.0	5.4	1.9–12.5
	Peak LH (mIU/L)		3.6			
	Basal FSH (mIU/L)		1.2	4.8	19.8	2.5–10.2
	Peak FSH (mIU/L)		9.5			
	Tesosterone (nmol/L)				22.0	8.4–28.8

A blood sample was collected for genetic analysis after obtaining parental informed consent with approval of the Institutional Review Board of Ha'Emek Medical Center. Additional, informed consent was obtained from the parents for publication of this case report and any potentially identification images/information. The exons and flanking intronic boundaries of the *POU1F1* gene were amplified and sequenced directly using primer sets that are available upon request. A homozygous frameshift mutation, c.580_581insT p.(Thr194Ilefs^*^7), formerly c.502insT (p.Thr168IlefsX7) as previously described by our group (30), was identified. This insertion results in a frameshift that introduces an early termination codon, leading to a severely truncated protein lacking the entire homeodomain. Both parents were heterozygous for the identified mutation. There is no known familial connection to the patient in whom this mutation was previously described.

## Discussion

We report a 17-year-old male with untreated CPHD (GH, TSH, and PRL deficiencies), who presented with extreme short stature, deaf-mutism, and neurological disabilities (both cognitive and physical) with a homozygous *POU1F1* mutation c.580_581insT p.(Thr194llefs^*^7). CPHD is classically divided into two subgroups according to the transcription factor gene types affected: early expressed pituitary transcription factor genes, defined as syndromic CPHD, and late expressed pituitary developmental genes, defined as non-syndromic CPHD. Whereas, mutations in early developmental genes are commonly involved in organogenesis and therefore present clinically with brain, ophthalmic, and/or skeletal anomalies, mutations in late developmental genes generally present with isolated pituitary hormone deficiencies in the absence of other anomalies (3). *POU1F1* mutations belong to the non-syndromic CPHD subgroup. Patients with *POU1F1* gene mutations are generally characterized by GH, PRL, and TSH deficiencies, without extrapituitary abnormalities (1–5). Variability exists in time of onset and severity of hormonal deficiencies; whilst GHD commonly presents in the neonatal period or early childhood, hypothyroidism may not occur at all or may present at any time from the neonatal period to the second decade of life. *POU1F1* mutations may in fact present with isolated GHD (28).

Neonatal thyroid screening in Israel aims to identify primary congenital hypothyroidism (CH), such that only abnormally elevated levels of TSH are reported. Central hypothyroidism (in which TSH is low) is thus not reported on neonatal screening, potentially resulting in delayed diagnosis. Furthermore, TSH is also frequently relied upon for screening when hypothyroidism is suspected later in life (with FT4 measured only if it is elevated), such that a diagnosis of central hypothyroidism may again be missed. In this case, diagnosis was delayed until 6 months of age with limited subsequent treatment compliance. LT_4_ therapy was ceased within 2 months of the initial diagnosis, briefly reinitiated at 5 years of age, but ceased again shortly thereafter until presentation to our clinic at age 17.5 years.

Descriptions of patients with CH before the widespread implementation of neonatal screening in the 1980s, as well as reports of children with endemic cretinism due to iodine deficiency, describe clinical phenotypes with severe cognitive and neurological impairment including spasticity, particularly in the lower extremities, shuffling gait, discoordination, jerky movements, tremor, hypotonia, and extrapyramidal disorders (33). In addition, deaf-mutism, hearing loss, dysarthria, and extreme short stature are reported (33, 34). It is therefore likely that the cognitive impairment, hypotonia, neurological deficits, and deaf-mutism seen in this case are consequences of delayed diagnosis and poor compliance with LT_4_ treatment. Mental retardation, microcephaly, sensorineural deafness, and severe prenatal and postnatal growth failure were described in patients with IGF-1 mutation (35) but not in patients with IGF-1 deficiency secondary to GH deficiency despite the occurrence of recurrent hypoglycemic events in infancy. This may indicate that the severe mental retardation in our case is attributable primarily to prolonged untreated hypothyroidism rather than unrecognized hypoglycemic events in infancy. Early diagnosis and initiation of LT_4_ therapy as well as GH treatment might have prevented neurodevelopmental deterioration and improved final height. No previous descriptions of *POU1F1* mutations describe the untreated natural history of this condition. This case therefore extends the phenotypic spectrum associated with *POU1F1* mutations, highlighting the importance of appropriate treatment and follow-up.

Although GH therapy resulted in a 42 cm (+3 SD) height increment, final height was only 124 cm (−6.2 SD). In our previously reported patient with the same *POU1F1* mutation, GH therapy was initiated in the first year of life but final height was only 154 cm (−3.15 SD) (30). Whilst in the latter case, there may also have been difficulties with treatment compliance, severe adult short stature has been reported in other subjects with *POU1F1* mutations [131 cm (24), 119 cm (21), and 116 cm (27)].

Sequencing of the candidate gene, *POU1F1*, revealed a homozygous mutation c.580_581 insT p.(Thr194llefs^*^7) and heterozygous parents. This mutation abolishes the transactivation properties of POU1F1 on three target promoters, resulting in severe TSH, PRL and GH deficiencies (30) which may be additional cause for the extreme short final height in the proband.

## Conclusion

This case of a 17-year-old male with a frameshift mutation in the *POU1F1* gene illustrates the consequences of inadequate CPHD treatment. The remarkable presentation of this patient, who looked 2 years old at age 17.5 years, and suffered from deafness and extensive neurological impairment, extends the recognized phenotype of *POU1F1* mutations and emphasizes the great importance of early and adequate thyroid and GH supplemental therapy.

## Data Availability

No datasets were generated or analyzed for this study.

## Ethics Statement

This study was approved by the Institutional Review Board of Ha'Emek Medical Center. The patient is described anonymously and gave written informed consent with the publication.

## Author Contributions

All authors listed have made a substantial, direct and intellectual contribution to the work, and approved it for publication.

### Conflict of Interest Statement

The authors declare that the research was conducted in the absence of any commercial or financial relationships that could be construed as a potential conflict of interest.
